# Elevated Expression of *RGS2* May Underlie Reduced Olfaction in COVID-19 Patients

**DOI:** 10.3390/jpm12091396

**Published:** 2022-08-28

**Authors:** Eden Avnat, Guy Shapira, David Gurwitz, Noam Shomron

**Affiliations:** 1 Faculty of Medicine, Tel Aviv University, Tel Aviv 69978, Israel; 2 Edmond J Safra Center for Bioinformatics, Tel Aviv University, Tel Aviv 69978, Israel; 3 Sagol School of Neuroscience, Tel Aviv University, Tel Aviv 69978, Israel

**Keywords:** anosmia, COVID-19, SARS-CoV-2, *RGS2*, NAMPT, PTGS2, CXCL8, RNA-sequencing, nasopharyngeal epithelial cells

## Abstract

Anosmia is common in COVID-19 patients, lasting for weeks or months following recovery. The biological mechanism underlying olfactory deficiency in COVID-19 does not involve direct damage to nasal olfactory neurons, which do not express the proteins required for SARS-CoV-2 infection. A recent study suggested that anosmia results from downregulation of olfactory receptors. We hypothesized that anosmia in COVID-19 may also reflect SARS-CoV-2 infection-driven elevated expression of regulator of G protein signaling 2 (*RGS2*), a key regulator of odorant receptors, thereby silencing their signaling. To test our hypothesis, we analyzed gene expression of nasopharyngeal swabs from SARS-CoV-2 positive patients and non-infected controls (two published RNA-sequencing datasets, 580 individuals). Our analysis found upregulated *RGS2* expression in SARS-CoV-2 positive patients (FC = 14.5, Padj = 1.69 × 10^−5^ and FC = 2.4; Padj = 0.001, per dataset). Additionally, *RGS2* expression was strongly correlated with *PTGS2, IL1B, CXCL8, NAMPT* and other inflammation markers with substantial upregulation in early infection. These observations suggest that upregulated expression of RGS2 may underlie anosmia in COVID-19 patients. As a regulator of numerous G-protein coupled receptors, RGS2 may drive further neurological symptoms of COVID-19. Studies are required for clarifying the cellular mechanisms by which SARS-CoV-2 infection drives the upregulation of *RGS2* and other genes implicated in inflammation. Insights on these pathway(s) may assist in understanding anosmia and additional neurological symptoms reported in COVID-19 patients.

## 1. Introduction

Deficiency or complete loss of smell (anosmia) is common in COVID-19, affecting a substantial number of patients and sometimes lasting for weeks or months following recovery [1,2]. Anosmia in COVID-19 patients was suggested as predictor for post-COVID-19 fatigue syndrome (“long COVID”; [3]) and observed as a comorbidity with persistent post-COVID inflammation [4]. The biological pathways underlying olfactory deficiency in COVID-19 remain unclear. Albeit SARS-CoV-2 may enter the brain [5,6], it does not seem to be capable of infecting olfactory neurons [7]. It was recently shown that SARS-CoV-2 infection causes downregulation of olfactory receptors in olfactory neurons, which may explain COVID-19 related anosmia [8]. We hypothesize that the olfactory dysfunction in COVID-19 may additionally reflect an infection-driven upregulation of *RGS2* (regulator of G protein signaling 2), a key regulator of nasal cavity G protein-coupled odorant receptors, whose signaling is diminished by the RGS2 protein [9]. RGS2 is known to be co-expressed with odorant receptors, and injection of RGS2 antibody into olfactory neurons was shown to enhance calcium currents in olfactory neurons stimulated with odorants [10]. Here, we present findings from analysis of RNA-sequencing data from nasopharyngeal samples, which show higher *RGS2* mRNA levels in COVID-19 patients. While the biological pathways upregulating *RGS2* mRNA in SARS-CoV-2 positive nasal tissues remain to be established, we present evidence that this is a consequence of the acute inflammation caused early in the span of infection. We present additional evidence for a correlation for higher RGS2 expression in populations with increased risk of COVID-19-induced anosmia.

## 2. Methods

We analyzed RNA-sequencing data from two NCBI Gene Expression Omnibus (GEO) database: GSE163151 [11] and GSE152075 [12] to comparing the expression of *RGS2* in SARS-CoV-2 positive and negative nasopharyngeal epithelial cells. For this analysis we applied a negative binomial generalized linear model under the standard DESeq2 method [13]. Significant findings were reported for Padj < 0.05. Additionally, Spearman correlation was calculated between genes for identifying genes with significant correlations with *RGS2* expression levels. Enrichment analysis was performed using Gene Ontology enRIchment anaLysis and visuaLizAtion tool (Gorilla) [14]. Figures were plotted using GraphPad Prism version 9.3.1 for Windows, GraphPad Software, La Jolla, CA, USA.

## 3. Results

**Figure 1:**
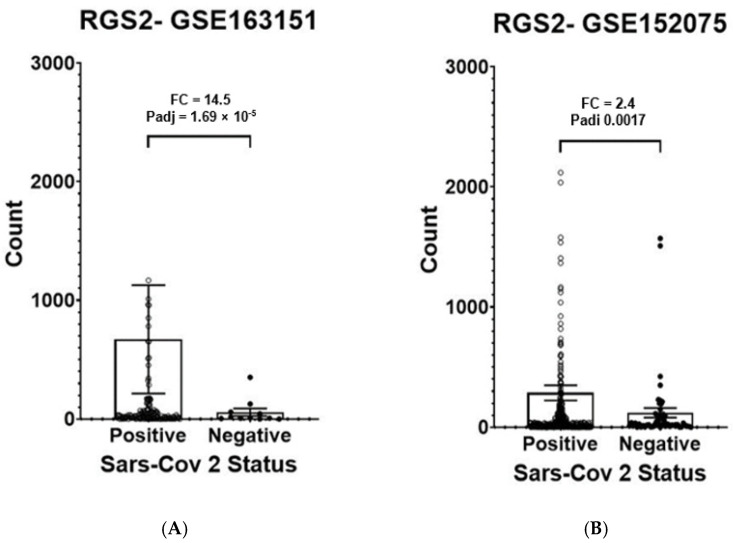
Expression levels of *RGS2* mRNA from RNA-sequencing of nasopharyngeal swabs of SARS-CoV-2 positive patients and negative controls. (**A**) Dataset GSE163151: higher expression of *RGS2* (FC = 14.5, padj = 1.69 × 10^−^^5^) in SARS-CoV-2 positive patients (138 samples) vs. negative controls (11 samples). (**B**) Dataset GSE152075: higher expression of *RGS2* (FC = 2.4, padj = 0.0017) in SARS-CoV-2 positive patients (377 samples) vs. negative controls (54 samples). Samples with over 3000 *RGS2* counts (five and nine SARS-CoV-2 positive patients, respectively) are not shown due to scale limitations but are included in the statistics.

**Figure 2:**
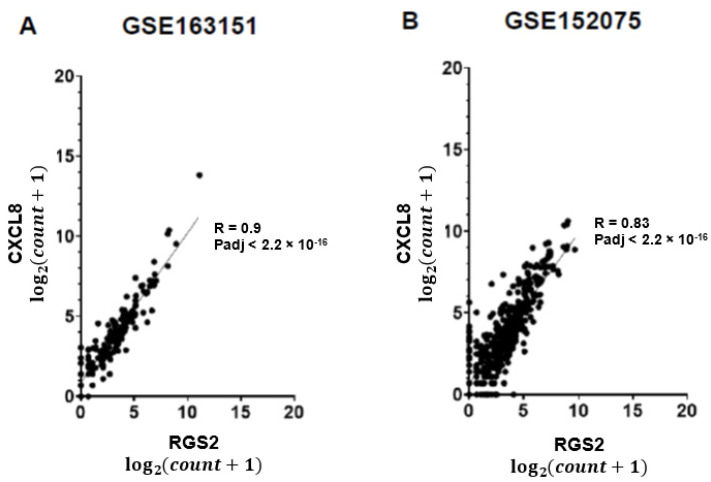
Positive correlation between expression levels of *RGS2* and *CXCL8* from RNA-sequencing of nasopharyngeal swabs. Spearman correlations are shown for (**A**) dataset GSE163151; (**B**) dataset GSE152075.

**Figure 3:**
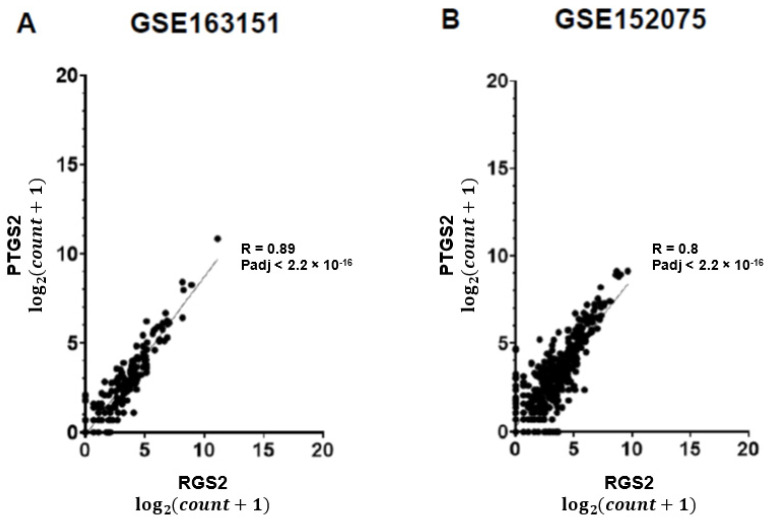
Positive correlation between expression levels of *RGS2* and *PTGS2* from RNA-sequencing of nasopharyngeal swabs. Spearman correlations are shown for (**A**) dataset GSE163151; (**B**) dataset GSE152075.

**Figure 4:**
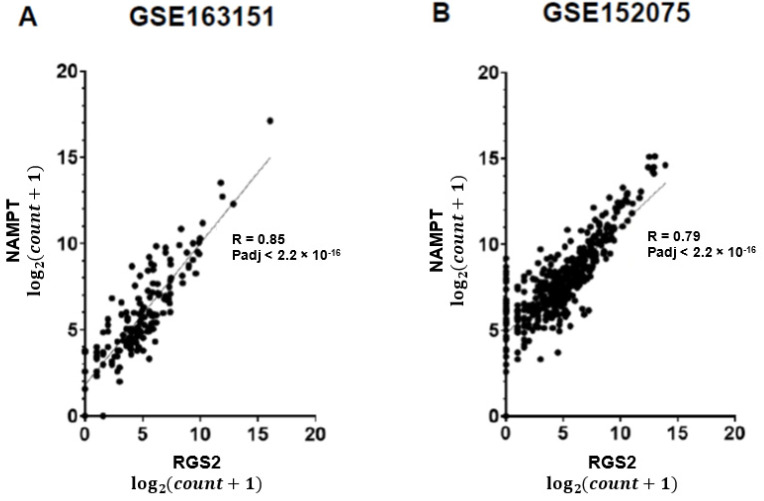
Positive correlation between expression levels of *RGS2* and *NAMPT* from RNA-sequencing of nasopharyngeal swabs. Spearman correlations are shown for (**A**) dataset GSE163151; (**B**) dataset GSE152075.

**Figure 5:**
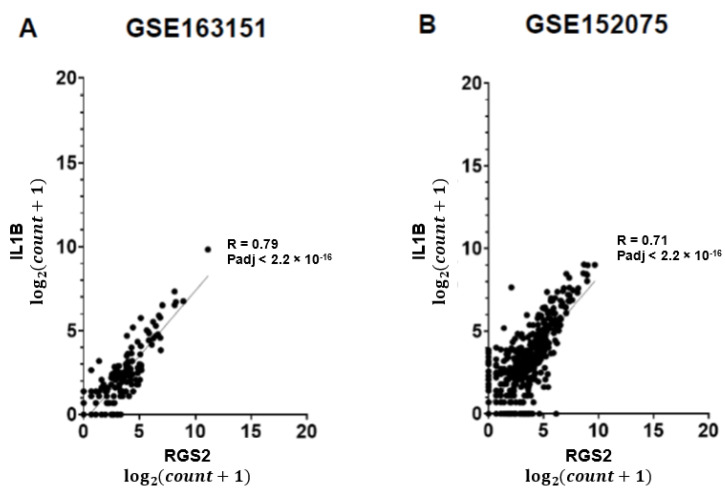
Positive correlation between expression levels of *RGS2* and *ILB1* from RNA-sequencing of nasopharyngeal swabs. Spearman correlations are shown for (**A**) dataset GSE163151; (**B**) dataset GSE152075.

We observed a 14.5-fold increase of *RGS2* mRNA expression in nasopharyngeal swabs of COVID-19 patients, compared with controls in both datasets (Padj = 1.7 × 10^−5^; Figure 1A and FC = 2.4; Padj = 0.001693; Figure 1B). The expression of RGS2 correlated with multiple genes many of which were associated with the immune signaling and inflammation observed in COVID-19. *CXCL8* (C-X-C motif chemokine ligand 8), which codes for interleukin-8, had the strongest correlation with *RGS2* (R = 0.91, *p* < 2.2 × 10^−16^ and R = 0.83, *p* < 2.2 × 10^−16^; Figure 2), followed by *PTGS2* (R = 0.89, *p* < 2.2 × 10^−16^; R = 0.8; *p* < 2.2 × 10^−16^; Figure 3), *NAMPT* (R = 0.85, *p* < 2.2 × 10^−16^; R = 0.79; *p* < 2.2 × 10^−16^; Figure 4) and *ILB1* (R = 0.79, *p* < 2.2 × 10^−16^; R = 0.71; *p* < 2.2 × 10^−16^; Figure 5).

The set of genes most correlated with *RGS2* expression (*p* < 2 × 10^−16^; Table 1) was significantly enriched for cellular response to interleukin-8, neutrophil aggregation and G protein-coupled receptor activity.

## 4. Discussion

We detected significant upregulation of *RGS2* mRNA expression in SARS-CoV-2 positive nasopharyngeal swabs, confirmed by two independent NCBI GEO datasets (Figure 1). RGS2 (regulator of G protein signaling 2) is recognized as the key regulator odorant receptor signaling [9]. Moreover, RGS2 is co-expressed with nasal odorant receptors, and odorant receptor-mediated calcium currents were enhanced by injection of RGS2 antibody into olfactory neurons [10]. Our findings therefore suggest that the elevated *RGS2* expression in SARS-CoV-2 positive nasopharyngeal cells is implicated in the common anosmia or reduced olfaction observed in many COVID-19 patients.

Anosmia is notable for being among the earliest symptoms to arise following SARS-CoV-2 infection [1,2,3,15]. *RGS2* expression is upregulated already during the first hours following SARS-CoV-2 infection, along with other early response genes, such as *PTGS2* [8,16].

COVID-19 associated anosmia is significantly more prevalent in young females [17], which are the demographic group with the highest *RGS2* blood expression [18]. Anosmia is most common among milder COVID-19 cases, which are also characterized as having higher levels of blood *RGS2* expression compared to more severe patients [17,18].

We hypothesize that COVID-19 associated anosmia is caused by the strong, acute nasal inflammation elicited by interferon in mild COVID-19 cases [19]. We suggest that *RGS2* upregulation is caused by inflammation of olfactory neurons, triggered via glial activation and IL-1-beta signaling, as previously observed in mouse models of intranasal lipopolysaccharide mediated inflammation [20].

A recent study of SARS-CoV-2 infection in model animals suggested that anosmia results from disruption of nuclear architecture [8]. This study further corroborates the indirect inflammation hypothesis, detecting significant nasal IL-1-beta and RGS2 upregulation in the first hours of infection [8].

Another potential clue for the mechanism underlying COVID-19 associated anosmia could be derived from the recent emergence of the SARS-CoV-2 Omicron variants, which was accompanied by a drastic reduction of anosmia cases among British COVID-19 patients from 52.7% during the Delta wave to 16.7% during the Omicron wave [21]. Notably, the NCBI GEO datasets applied for our study were generated from COVID-19 patients prior to the emergence of the Omicron variants. Consistent with our hypothesis, we postulate that the reduced immunogenicity of the Omicron variant leads to a significantly milder initial immune response [22] and in turn, milder induction of *RGS2* expression and thus reduced risk of anosmia. This hypothesis is supported by the significantly milder upregulation of IL-1-beta and other primary immunogenic factors, in response to infections of mice with Omicron (both BA.1 and BA.2 subclades) compared to earlier SARS-CoV-2 strains [23]. The relation between early viral load and anosmia was previously observed [24,25].

As a regulator of several G-protein coupled receptors, the elevated expression of RGS2 may drive further neurological symptoms observed in COVID-19 patients, sometimes lingering beyond negative SARS-CoV-2 PCR test findings [26]. The highly correlated expression of *RGS2* with *CXCL8, PTGS2, NAMPT, ILB1* and further inflammatory genes in COVID-19 positive nasopharyngeal swabs (Figure 1, Figure 2, Figure 3, Figure 4 and Figure 5, Table 1) suggests the involvement of a common regulator, which might explain the distinct classes of COVID-19 symptoms. Changes in the expression of circulating or exosomal microRNAs in COVID-19 are one factor that might explain at least some of the observed gene expression alterations [27,28].

Our hypothesis has several limitations. Our findings on higher *RGS2* mRNA levels in nasopharyngeal samples from COVID-19 patients compared with controls are based on data mining of published NCBI GEO datasets. Therefore, we could not validate our findings by real-time qPCR experiments or by measuring RGS2 protein levels. Additionally, the COVID-19 severity and the anosmia status in these COVID-19 patients are unknown. As the two GSE datasets applied for our study were published by two different research teams, we estimated the variance between the datasets using DESeq2 covariates. This yielded a homogenous merged dataset, thus reducing concerns on team-specific variation in swab collection protocols.

Further studies of COVID-19 animal models are required for clarifying the cellular mechanisms by which SARS-CoV-2 infection drives higher expression of *RGS2* mRNA. Insights into the pathways driving anosmia in COVID-19 may improve our understanding of additional neurological indications reported in these patients [29].

## Figures and Tables

**Table 1:** List of genes with expression levels correlations with *RGS2* in nasopharyngeal swabs from SARS-CoV-2 positive and negative individuals. Genes in GSE163151 and GSE152075 found with mRNA expression correlated with *RGS2* expression (with R > 0.75 in at least of these GSE files) are listed by decreasing R values for GSE152075. All correlations had *p* < 2.2 × 10^−16^. See Methods for further information.

Gene	GSE163151	GSE152075
*CXCL8*	0.9066	0.8300
*PTGS2*	0.8875	0.8043
*AQP9*	0.8853	0.7913
*NAMPT*	0.8467	0.7900
*MXD1*	0.7933	0.7795
*SRGN*	0.8550	0.7619
*NCF2*	0.7731	0.7402
*BTG2*	0.8292	0.7357
*MNDA*	0.8557	0.7314
*RNF149*	0.7677	0.7213
*ILB1*	0.7938	0.7140

## Data Availability

The data in this article were derived from the following NCBI GEO resources: GSE163151 (https://www.ncbi.nlm.nih.gov/geo/query/acc.cgi?acc=GSE163151 accessed on 18 February 2022); and GSE152075 (https://www.ncbi.nlm.nih.gov/geo/query/acc.cgi?acc=GSE152075, accessed on 19 February 2022). See Methods for further information.

## Abbreviations

ACE2	angiotensin-converting enzyme 2
COVID-19	coronavirus disease-2019
CXCL8	C-X-C motif chemokine ligand 8
GEO	gene expression omnibus
IL1B	interleukin 1 beta
NAD	nicotinamide adenine dinucleotide
NAMPT	nicotinamide phosphoribosyltransferase
NCBI	National Center for Biotechnology Information
PTGS2	prostaglandin-endoperoxide synthase 2
RGS2	regulator of G protein signaling 2
SARS-CoV-2	severe acute respiratory syndrome coronavirus 2
TMPRSS2	transmembrane serine protease 2
